# Interactions of Six SNPs in ABCA1gene and Obesity in Low HDL-C Disease in Kazakh of China

**DOI:** 10.3390/ijerph13020176

**Published:** 2016-01-28

**Authors:** Ming-hong Yao, Heng Guo, Jia He, Yi-zhong Yan, Ru-lin Ma, Yu-song Ding, Jing-yu Zhang, Jia-ming Liu, Mei Zhang, Shu-gang Li, Shang-zhi Xu, Qiang Niu, Jiao-long Ma, Shu-xia Guo

**Affiliations:** Department of Public Health and Key Laboratory of Xinjiang Endemic and Ethnic Diseases of the Ministry of Education, Shihezi University School of Medicine, Shihezi 832002, China; ymhldjxa@sina.com (M.-H.Y.); guoheng@shzu.edu.cn (H.G.); hejia123.shihezi@163.com (J.H.); erniu19880215@sina.com (Y.-Z.Y.); marulin@126.com (R.-L.M.); 13399931625@163.com (Y.-S.D.); yfyxxzjy@126.com (J.-Y.Z.); liujiaming@shzu.edu.cn (J.-M.L.); zmberry@foxmail.com (M.Z.); lishugang@ymail.com (S.-G.L.); yfyxxxsz@sina.com (S.-Z.X.); niuqiang1214@163.com (Q.N.); jiaojiaolong881202@163.com (J.-L.M.)

**Keywords:** ABCA1, obesity, interaction, low HDL-C, Kazakh

## Abstract

*Objective*: To detect the interactions between six functional polymorphisms in ABCA1 and obesity in Kazakhs with low HDL-C levels. *Methods*: A total of 204 patients with low HDL-C and 207 health control subjects, which were randomly selected from among 5692 adult Kazakhs, were matched for age and sex. We genotyped ABCA1 single nucleotide polymorphisms of rs2515602, rs3890182, rs2275542, rs2230806, rs1800976, and rs4149313. *Results*: (1) The genotypic and allelic frequencies of rs2515602, rs2230806 and rs4149313 were different between normal HDL-C and low HDL-C subjects, the genotypic frequency of rs2275542 was also different between normal HDL-C and low HDL-C subjects (*p* < 0.05); (2) the level of HDL-C (rs2515602 and rs2275542) in normal HDL-C subjects were different among the genotypes (*p* < 0.05); the levels of TC, LDL-C (rs2515602, rs4149313); TG (rs2515602, rs1800976, rs4149313) in low HDL-C patients were different among the genotypes (*p* < 0.05); (3) interactions between the rs3890182, rs2275542, rs180096, and rs4149313 polymorphisms in ABCA1 gene and obesity may be associated with low HDL-C disease; (4) the C-C-C-A-A-G, T-C-C-A-A-A, T-C-C-A-A-G, C-C-C-A-A-A, C-T-G-G-A-A, and T-T-C-G-A-A haplotypes were significant between the subjects with normal HDL-C and low HDL-C level (*p* < 0.05). *Conclusions*: The differences in serum lipid levels between normal HDL-C and low HDL-C subjects among Kazakhs might partly result from ABCA1 gene polymorphisms; ABCA1 gene polymorphisms may be associated with low HDL-C disease; the low HDL-C disease might partly result from interactions between ABCA1 gene polymorphisms and obesity; the C-C-C-A-A-G, T-C-C-A-A-A, and T-C-C-A-A-G haplotypes may serve as risk factors of low HDL-C disease among Kazakhs, the C-C-C-A-A-A, C-T-G-G-A-A, and T-T-C-G-A-A haplotypes may serve as protective factor of low HDL-C disease among Kazakhs.

## 1. Introduction

Dyslipidemia has become one of the most serious public health problems in the world because of its high prevalence [1,2] and a causal relationship of conditions such as coronary artery disease (CAD) [3], obesity and metabolic syndrome [4]. Low high-density lipoprotein-cholesterol (HDL-C) as one of the important phenotype of dyslipidemia and it shows higher prevalence than other three kinds of components in dyslipidemia [5,6]. Low HDL-C damage to health is mainly concentrated in the cardiovascular system. A large body of epidemiological evidence has shown a strong inverse relationship between serum levels of HDL-C and CAD [7,8]. HDL-C was demonstrated to play a pivotal role in mediating the transfer of cholesterol from extrahepatic tissues to the liver [9,10]. 

The serum levels and function of HDL-C are influenced by genetic and environmental factors as well as their interactions [11]. ATP-binding cassette transporter A1 (ABCA1) gene, consisting of 49 exons, is located on chromosome 9q31, and encodes the key protein that directs the excessive efflux of lipids from peripheral cells into lipid-poor apolipoprotein A1 particles and facilitates the formation of HDL-C [12,13,14]. ABCA1 gene mutation may affect the transcription and expression of the protein, thereby affecting serum HDL-C level [15,16]. Besides, the link between dyslipidemia and obesity has been well documented [17]. Obesity not only affects the quality of the HDL-C, but also affects the concentration of HDL-C in serum [18,19,20,21]. In recent years, numerous studies have shown a relationship between the ABCA1 gene polymorphism and HDL-C, however, this still remain inconsistent across different races [11,22,23,24,25,26,27]. A major reason for the inconsistency among studies may be interactions between different environmental factors and genes that influence serum HDL-C levels. 

There are studies showing a relationship between ABCA1 gene and the HDL-C concentration in obese populations [28,29]. However, the regulation of ABCA1 on level of HDL-C in obesity has not been clearly defined yet. Based on the above observations, we hypothesized that low HDL-C disease might partly result from interactions between ABCA1 gene polymorphisms and obesity. The Kazakh nationality in Xinjiang (China) has a unique culture and customs, and the prevalence of obesity and low HDL-C disease are higher than that of the Han nationality in the same area [6,30]. To our knowledge, the relationship between serum lipid levels and ABCA1 gene polymorphisms in Kazakhs has not been reported. We therefore investigated six (rs2515602, rs2275542, rs2230806, rs1800976, rs4149313, rs3890182) single nucleotide polymorphisms (SNPs) in the ABCA1 gene in a sample consisting of 411 (low HDL-C: 204 and normal control: 207) Kazakh individuals to detect the risk of the interaction ABCA1 gene polymorphisms-obesity and ABCA1 gene variants in low HDL-C disease. In addition, we also studied the relationship between ABCA1 gene variants and serum lipids.

## 2. Methods

### 2.1. Study Population

The subjects in this study consisted of 411 unrelated adults who resided in Xinyuan County and Jiashi County, Xinjiang Uyghur Autonomous Region, People’s Republic of China. They were randomly selected from our previous stratified randomized cluster samples [30]. Two hundred and four (204) patients with low HDL-C disease were randomly selected as the case group and 207 normal subjects who had no evidence of liver diseases, renal diseases, or malignant tumors were randomly selected as the control group using the group-matching method. The subjects were not taking medications known to effect plasma lipid levels. The protocol was approved by the Institutional Ethics Review Board (IERB) of the First Affiliated Hospital of Shihezi University School of Medicine (IERB No. SHZ2010LL01). Witten informed consent was obtained from each participant.

### 2.2. Epidemiological Survey and Diagnostic Criteria

Information on demographic and personal lifestyles was collected with a self-developed questionnaire during face-to-face interviews. Blood pressure, height, weight, waist circumference and hip circumference were measured according to standardized methods [31]. Body mass index (BMI) was calculated by weight (kg) divided by the square of measured height (m^2^). The diagnostic criteria of obesity and normal weight were defined as a BMI > 28, and <24 kg/m^2^; respectively [32]. High TG, low HDL-C, high LDL-C, and high TC were defined as follows: TG ≥ 1.70 mmol/L; (2) HDL-C < 1.04, mmol/L; (3)LDL-C ≥ 2.59 mmol/L; (4) TC ≥ 5.17 mmol/L; respectively [33]. Diabetes was defined as FPG ≥ 7.0 mmol/L [34].

### 2.3. Biochemical Measurements

After overnight fasting, venous blood samples (5 mL) were drawn from the forearm vein of all participants. A part of the blood (3 mL) was collected into glass tubes and used to determine the serum lipid levels. Another part of the blood (2 mL) was transferred into tubes and used to extract deoxyribonucleic acid (DNA). The concentrations of triglycerides (TGs), total cholesterol (TC), HDL-C, fasting plasma glucose (FPG), and low density lipoprotein cholesterin (LDL-C) in serum were measured by using a DXC-800 automatic biochemical analyzer (Beckman, Pasadena, CA, USA) located at the Clinical Science Experiment Center of the First Affiliated Hospital of Shihezi University School of Medicine.

### 2.4. DNA Extraction

Fasting venous blood (200 µL) was taken from each study subject and a blood genomic DNA isolation kit (Non-centrifugal columnar, Tiangen, Beijing, China) was used to extract the whole blood genomic DNA. The extracted DNA was verified by gel electrophoresis (0.7% agarose). A NanoDrop spectrophotometer (Thermo Scientific, Waltham, MA, USA) was used for quantitative determination of DNA concentration and purity: concentration ≥30 ng/µL and purity levels (OD260/OD280) of 1.7–2.0 were considered acceptable. Samples that met these criteria were diluted to 10–30 ng/µL using double-distilled water and were stored at −80 °C.

### 2.5. PCR Amplification

Primers were designed using the Mysequenom tool (www.mysequenom.com/Home) and AssayDesigner3.0 software (Sequenom, Inc., San Diego, CA, USA). The sequences of the forward and reverse primers used for the genotyping of six SNPs are list in Table 1. Final PCR reaction volumes were 15 µL, which included 1 µL DNA samples, 0.3 µL dNTPs, 7.4 µL water, 1.5 μL 10 × PCR buffer, 1.5 µL MgCl_2_, 0.3 µL Taq enzymes, 3 μL mixture of PCR amplification primers. Cycling conditions were as follows: predegeneration at 94 °C for 4 min; followed by 35 cycles of denaturation at 94 °C for 20 s, annealing at 56 °C for 30 s, and extension at 72 °C for 1 min. A final extension step was carried out at 72 °C for 3 min, after which samples were maintained at 4 °C. Reactions were set up in an ice bath and each PCR experiment included a negative control reaction.

### 2.6. PCR Product Purification

Shrimp alkaline phosphatase (SAP) was used to remove excess dNTPs from samples after PCR. This step served to ensure the accuracy of single-base extension. The final SAP reaction volumes were 5.0 mL, which included 0.5 µL 10× SAP buffer, 2 uL PCR product, 2 µL double-distilled water, and 0.5 µL SAP enzyme. Reactions were conducted by incubation at 37 °C for 40 min, followed by incubation at 85 °C for 5 min. The reaction products were stored at 4 °C.

### 2.7. Single-Base Extension

For single-base extension reactions, final reaction volumes were 6.0 µL, which included 0.5 μL Snapshot reagent, 2.5 µL water, 1 µL primer mix, 2 uL PCR products purified. Reaction conditions were as follows: denaturation at 94 °C for 30 s; followed by 40 cycles of 94 °C for 5 s, 52 °C for 5 s, and finally 52 °C for 5 s. Reaction products were stored at 4 °C.

### 2.8. Genotyping Analysis

Take 1 µL reaction product plus 9 µL HIDI, 95 °C denaturation 3 min, immediately ice-water bath, All representative SNP genotyping experiments were done using TaqMan technology on an ABI3730XL system (Applied Biosystems, Foster City, CA, USA). Genemapper was used to complete the classification and present the results.

**Table 1:** The sequences of forward and reverse primers for genotyping of the ABCA1 gene.

SNP	Forward Sequence	Reverse Sequence	PCR Product	Allele
rs2515602	5’-CAGTGAAAACAATGGTGAGGC-3’	5’-CATCTATGTGGAGAGATGTGG-3’	235bp	A/G
rs3890182	5’-AAGAACACTCGCAAAGTCAGC-3’	5’-TGTGTTTTTCAGGTGCCCTTG-3’	208bp	C/T
rs2275542	5’-AATGCAGTTGGCAGCAATCTG-3’	5’-TCCCATTAGATCTTCCCCAAG-3’	208bp	A/G
rs2230806	5’-CTTGTGCTTGTCTCTCTTTGC-3’	5’-ATTGGCTTCAGGATGTCCATG-3’	237bp	C/G
rs1800976	5’-GGAACGTGGACTAGAGAGTCTG-3’	5’-AGTCACTCAGCAGAAAGCACG-3’	216bp	C/T
rs4149313	5’-TGGGAAACCCTCAGAATACTG-3’	5’-GTTAGCAGAGGCAGCAGCACTA-3’	210bp	A/G

### 2.9. Statistical Analysis

Epidata 3.02 software (EpiData Association, Odense, Denmark) was used to establish a database, and the double entry method was used for data input and logic error detection. Non-normally distributed continuous variables such as TG, TC, HDL-C, and LDL-C are shown as median and interquartile range (25th, 75th percentile), while age, height, weight, BMI, waist circumference, hip circumference, waist-to-hip ratio, systolic blood pressure, diastolic blood pressure, pulse pressure, and FPG are presented as mean ± standard deviation. Categorical variables are shown as frequencies or percentages. The Kruskal-Wallis H statistic or One Way ANOVA was used to compare continuous variables among the three genotype groups, while the Kolmogorov-Smirnov Z test or the Student’s *t*-Test. The frequency of the ABCA1 alleles was determined by gene counting. Chi square tests were used to compare the differences in percentages and to assess Hardy-Weinberg equilibrium. Interactions between six SNPs in ABCA1 gene and obese were assessed by using unconditioned Logistic regression analysis after controlling for potential confounders which included sex, age, hypertension, high TC, high TG, high LDL-C, smoker, drinker and diabetes. SHEsis software was used to analysis haplotype [35]. Frequency table and statistical analysis were used with the SPSS 17.0 (SPSS, Inc. Chicago, IL, USA) statistical package. *p* = 0.05 was used to define the level of significance. 

## 3. Results

### 3.1. Clinical Data and Biochemical Characteristics of Study Subjects

Table 2 shows the clinical profiles of the participants. Weight, BMI, hip circumference, waist circumference, TG, FPG, and obesity prevalence were higher in the cases compared with controls (*p* < 0.05), whereas serum TC, LDL-C, and HDL-C levels were lower in the case group than in the control group (*p* < 0.05). 

**Table 2:** The general characteristics and serum lipid levels between the control group and case group.

Characteristics	Control (*n* = 207)	Case (*n* = 204)	*p*
Male/female	98/109	108/96	0.256
Age, years	41.01 ± 13.07	41.92 ± 13.75	0.409
Height, cm	162.96 ± 8.21	165.57 ± 8.50	0.002
Weight, kg	63.59 ± 11.91	71.43 ± 15.76	*p* < 0.001
Body mass index, kg/m^2^	23.95 ± 4.26	25.90 ± 4.62	*p* < 0.001
Waist circumference, cm	84.00 ± 11.95	89.23 ± 13.78	*p* < 0.001
Hip circumference	95.52 ± 7.46	98.27 ± 8.96	0.001
Waist-to-hip ratio	0.88 ± 0.08	0.90 ± 0.08	0.001
Obesity, *n* (%)	98 (47.34)	121 (59.31)	0.015
Systolic blood pressure, mmHg	135.98 ± 25.78	132.48 ± 22.83	0.146
Diastolic blood pressure, mmHg	85.23 ± 12.84	84.42 ± 14.40	0.550
Pulse pressure, mmHg	50.75 ± 18.35	48.06 ± 15.21	0.106
TC, mmol/L	4.20 (3.55–4.76)	3.68 (3.00–4.35)	*p* < 0.001
TG, mmol/L	0.83 (0.62–1.24)	1.12 (0.77–1.75)	*p* < 0.001
HDL-C, mmol/L	1.43 (1.31–1.63)	0.95 (0.84–0.99)	*p* < 0.001
LDL-C, mmol/L	2.06 (1.65–2.60)	1.93 (1.44–2.49)	0.021
FPG, (mmol/L)	4.49 ± 1.48	4.88 ± 1.49	0.009
Diabetic, *n* (%)	8 (3.86)	5 (2.45)	0.413
MI, *n* (%)	6 (2.90)	9 (4.41)	0.424
Smoker, *n* (%)	77 (37.20)	81 (39.71)	0.601
Alcohol drinker, *n* (%)	20 (9.66)	18 (8.82)	0.769

Notes: TG, triglyceride; TC, total cholesterol; LDL-C, low-density lipoprotein-cholesterol; HDL-C, high-density lipoprotein-cholesterol; FPG, fasting plasma glucose; MI, myocardial infarction.

There were no significance differences in values of male-to-female ratio, average age, systolic and diastolic blood pressure, pulse, diabetics, myocardial infarction, FPG, smokers, and alcohol drinkers between the control group and case group.

### 3.2. Hardy-Weinberg Equilibrium Testing of and Success Rate of Gene Frequencies

In our study, six SNPs in the ABCA1 gene were genotyped, and all loci were in agreement with Hardy-Weinberg equilibrium (all *p* > 0.05), indicating that the six loci of the ABCA1 gene reached genetic equilibrium and thus that the samples were indeed representative of the Kazakh nationality (Table 3). For the six SNPs, the success rates were all 100%.

**Table 3:** The genotypic and allelic frequencies between the subjects with normal HDL-C and low HDL-C.

SNPs	Control	Case	OR	95% CI	*p*
rs2515602					
CC *n* (%)	47 (22.7)	82 (40.2)	1		
CT *n* (%)	88 (42.5)	84 (41.2)	1.828	1.146–2.915	0.011
TT *n* (%)	72 (34.8)	38 (18.6)	3.306	1.942–5.627	*p* < 0.001
C allele *n* (%)	182 (43.9)	248 (60.8)	1.976	1.497–2.608	*p* < 0.001
HWE-P	0.377	0.35			
rs3890182					
GG *n* (%)	193 (93.2)	183 (89.7)	1		
AG *n* (%)	12(5.8)	21 (10.3)	0.542	0.259–1.133	0.099
AA *n* (%)	2 (1.0)	-	-	-	-
G allele *n* (%)	398 (96.1)	387 (94.9)	0.880	0.466 - 1.662	0.693
HWE-P	0.768	0.576			
rs2275542					
CC *n* (%)	110 (53.1)	87 (42.6)	1		
CT *n* (%)	74 (35.7)	96 (47.1)	0.610	0.403–0.922	0.019
TT *n* (%)	23 (11.1)	21 (10.3)	0.866	0.450–1.668	0.667
C allele *n* (%)	294 (71.0)	270 (66.2)	0.799	0.594–1.073	0.135
HWE-P	0.374	0.905			
rs2230806					
AA *n* (%)	20 (9.7)	44 (21.6)	1		
AG *n* (%)	89 (43.0)	98 (48.0)	1.998	1.095–3.646	0.023
GG *n* (%)	98 (47.3)	62 (30.4)	3.477	1.877–6.444	*p* < 0.001
A allele *n* (%)	129 (31.2)	186 (45.6)	1.851	1.392–2.461	*p* < 0.001
HWE-P	0.99	0.956			
rs1800976					
CC *n* (%)	41 (19.8)	50 (24.5)	1		
CG *n* (%)	114 (55.1)	91 (44.6)	1.528	0.930–2.510	0.093
GG *n* (%)	52 (25.1)	63 (30.9)	1.007	0.579–1.749	0.981
C allele *n* (%)	196 (47.3)	191 (46.8)	0.979	0.744–1.287	0.879
HWE-P	0.556	0.609			
rs4149313					
AA *n* (%)	99 (47.8)	64 (31.4)	1		
AG *n* (%)	84 (40.6)	94 (46.1)	0.578	0.376–0.889	0.012
GG *n* (%)	24 (11.6)	46 (22.5)	0.337	0.188–0.606	*p* < 0.001
A allele *n* (%)	282 (68.1)	222 (54.4)	0.559	0.421–0.742	*p* < 0.001
HWE-P	0.797	0.727			

Note: HWE-P, Hardy-Weinberg equilibrium *p* value. Rs2515602-CC, rs3890182-GG, rs2275542-CC, rs2230806-AA, rs1800976-CC, and rs4149313-AA genotypes used as a reference genotype for obtaining the Odds Ratio calculations separated for each single nucleotide polymorphism.

### 3.3. Genotype and Allele Frequencies

The genotype and allele frequencies of the six SNPs between normal HDL-C and low HDL-C are presented in Table 3. There were no significance differences in the genotype and allele frequencies of rs3890182 and rs1800976 between the control group and case group (*p* > 0.05). For the rs2515602 polymorphism, we observed a higher frequency of the C allele (60.8% *vs.* 43.9%, OR: 1.976; 95% CI: 1.497–2.608; *p* < 0.001) in low HDL-C patients compared with control group. Carriers with rs2275542 CC genotype had 0.610 times lower risk to get low HDL-C disease than those with CT genotype (OR for 95% CI: 0.403–0.922; *p* = 0.019). Compared with rs2230806 G allele, carriers with A allele were 1.851 times more likely to get low HDL-C disease (OR for 95% CI: 1.392–2.461; *p* < 0.001). Moreover, subjects who carried the rs4149313 A allele were significantly less likely to get low HDL-C disease (OR = 0.559, OR for 95% CI: 0.421–0.742; *p* < 0.001).

### 3.4. Correlation between Genotypes and Serum Lipid Profile between the Subjects with Normal HDL-C and Low HDL-C

Table 4 shows the correlation between the genotypes and serum lipid profiles between the subjects with normal HDL-C and low HDL-C. There are only two subjects with AA genotype in the control group according to rs3890182, so the levels of TC, TG, LDL-C, and HDL-C were not presented. Rs2515602 with CC genotype has lower HDL-C level than in with TT genotype in normal subjects, TT genotype has higher TG than in with CT/CC genotype and TT genotype has higher LDL-C and TC levels than in with CT genotype for cases (*p* < 0.05); Serum HDL-C level was higher in controls according to rs2275542 with TT genotype than in CC genotype (*p* < 0.05); Serum TG level was higher in cases according to rs1800976 with GG genotype than in CG genotype (*p* < 0.05); The level of TG was lower with AG/GG genotype than in with AA genotype and the levels of LDL-C and TC were higher with AA genotype than in with AG genotype in cases for rs4149313 (*p* < 0.05).

### 3.5. Interactions of the Six SNPs in ABCA1 Gene and Obesity in Patients with Low HDL-C

The interactions of six SNPs and obesity in patients with low HDL-C are presented in Table 5. Based on the adjustment of sex (1 = male, 2 = female), age (1 = 18–30, 2 = 31–40, 3 = 41–50, 4 = 50–60, 5 = 61~), hypertension (0 = no, 1 = yes), high TC (0 = no, 1 = yes), high TG (0 = no, 1 = yes ), high LDL-C (0 = no, 1 = yes), smoker (0 = no, 1 = yes), drinker (0 = no, 1 = yes) and diabetes (0 = no, 1 = yes ), the risk degree of interactions between obesity and the genotypes of six SNPs in ABCA1 gene (rs2515602: CC = 1, CT = 2, TT = 3; rs3890182: GG = 1, AG = 2, AA = 3; rs2275542: CC = 1, CT = 2, TT = 3; rs2230806: AA = 1, AG = 2, GG = 3; rs1800976: CC = 1, CG = 2, GG = 3; rs4149313: AA = 1, AG = 2, GG = 3) was evaluated by low HDL-C disease (0 = no, 1 = yes) were analyzed by logistic regression analysis. We found that there were interactions shown between rs3890182, rs2275542, rs1800976, and rs4149313 in ABCA1 gene and obesity in patients with low HDL-C.

**Table 4:** The genotypes of six SNPs and serum lipid levels (mmol/L) between the subjects with normal HDL-C and low HDL-C.

SNPs	Group	Genotype	*n* (%)	TG	TC	LDL-C	HDL-C
rs2515602	control	CC	47 (22.7)	0.81 (0.56–1.22)	3.98 (3.54–4.56)	2.06 (1.69–2.34)	1.35 (1.27–1.48) *
		CT	88 (42.5)	0.89 (0.64–1.70)	4.23 (3.63–4.79)	2.11 (1.74–2.73)	1.43 (1.31–1.59)
		TT	72 (34.8)	0.82 (0.62–1.11)	4.24 (3.55–4.80)	2.01 (1.62–2.62)	1.55 (1.36–1.71) *
	case	CC	82 (40.2)	1.10 (0.79–1.59) *	3.63 (3.07–4.31)	1.92 (1.46–2.52)	0.95 (0.87–0.99)
		CT	84 (41.2)	1.07 (0.71–1.54) *	3.63 (2.54–4.18) *	1.81 (1.27–2.38) *	0.95 (0.83–0.99)
		TT	38 (18.6)	1.46 (1.02–2.55) *	4.05 (3.24–4.77) *	2.11 (1.66–2.56) *	0.95 (0.86–0.99)
rs3890182	control	AA	193 (93.2)	0.83 (0.62–1.25)	4.23 (3.59–4.79)	2.08 (1.67–2.65)	1.43 (1.31–1.64)
		AG	12 (5.8)	0.77 (0.52–1.21)	3.83 (3.47–4.60)	1.95 (1.52–2.47)	1.42 (1.29–1.50)
	case	AA	183 (89.7)	1.14 (0.80–1.90)	3.76 (3.04–4.39)	1.96 (1.46–2.51)	0.95 (0.84–0.99)
		AG	21 (10.3)	0.90 (0.67–1.33)	3.29 (2.83–4.02)	1.66 (1.36–2.32)	0.97 (0.88–1.00)
rs2275542	control	CC	110 (53.1)	0.81 (0.61–1.38)	4.21 (3.49–4.85)	2.09 (1.64–2.72)	1.42 (1.30–1.60) *
		CT	74 (35.7)	0.85 (0.64–1.24)	4.22 (3.68–4.77)	1.99 (1.65–2.61)	1.42 (1.32–1.64)
		TT	23 (11.1)	0.82 (0.53–1.04)	4.16 (3.78–4.66)	2.07 (1.82–2.23)	1.56 (1.35–1.79) *
	case	CC	87 (42.6)	1.10 (0.75–1.90)	3.85 (3.08–4.44)	1.96 (1.39–2.54)	0.95 (0.87–1.00)
		CT	96 (47.1)	1.13 (0.81–1.57)	3.59 (2.84–4.13)	1.88 (1.44–2.38)	0.95 (0.84–0.98)
		TT	21 (10.3)	1.13 (0.78–2.32)	3.64 (3.05–4.99)	2.17 (1.66–2.80)	0.96 (0.84–1.00)
rs2230806	control	AA	20 (9.7)	0.81 (0.51–1.24)	4.33 (3.58–4.66)	1.94 (1.76–2.36)	1.36 (1.25–1.57)
		AG	89 (43.0)	0.84 (0.66–1.39)	4.20 (3.64–4.84)	2.16 (1.66–2.73)	1.43 (1.30–1.63)
		GG	98 (47.3)	0.83 (0.62–1.12)	4.16 (3.54–4.77)	2.01 (1.65–2.57)	1.46 (1.33–1.65)
	case	AA	44 (21.6)	1.09 (0.88–1.40)	3.59 (3.08–4.21)	1.89 (1.48–2.38)	0.95 (0.86–0.99)
		AG	98 (48.0)	1.11 (0.71–1.77)	3.68 (2.88–4.31)	1.90 (1.42–2.57)	0.95 (0.84–1.00)
		GG	62 (30.4)	1.39 (0.78–2.06)	3.91 (2.84–4.54)	1.98 (1.42–2.41)	0.95 (0.85–0.99)
rs1800976	control	CC	41 (19.8)	0.84 (0.63–1.42)	4.28 (3.38–4.79)	1.90 (1.54–2.79)	1.36 (1.28–1.85)
		CG	114 (55.1)	0.84 (0.58–1.27)	4.28 (3.80–4.70)	2.11 (1.71–2.64)	1.43 (1.32–1.59)
		GG	52 (25.1)	0.80 (0.58–1.06)	4.01 (3.43–4.77)	1.96 (1.55–2.44)	1.46 (1.31–1.67)
	case	CC	50 (24.5)	1.29 (0.81–2.04)	3.84 (2.97–4.92)	1.82 (1.44–2.65)	0.96 (0.85–1.00)
		CG	91 (44.6)	0.94 (0.73–1.41) *	3.63 (3.06–4.21)	1.91 (1.46–2.38)	0.93 (0.84–0.99)
		GG	63 (30.9)	1.25 (0.88–2.17) *	3.70 (2.85–4.52)	1.96 (1.38–2.56)	0.95 (0.86–0.99)
rs4149313	control	AA	99 (47.8)	0.83 (0.62–1.32)	4.23 (3.48–4.93)	2.00 (1.53–2.73)	1.48 (1.31–1.67)
		AG	84 (40.6)	0.84 (0.64–1.30)	4.18 (3.71–4.78)	2.15 (1.77–2.64)	1.40 (1.31–1.58)
		GG	24 (11.6)	0.77 (0.56–1.15)	4.04 (3.69–4.45)	1.89 (1.70–2.27)	1.38 (1.30–1.48)
	case	AA	64 (31.4)	1.46 (1.01–2.42) *	3.92 (3.16–4.64) *	2.07 (1.66–2.54) *	0.93 (0.84–0.99)
		AG	94 (46.1)	1.06 (0.70–1.46) *	3.63 (2.74–4.21) *	1.81 (1.36–2.40) *	0.96 (0.85–0.99)
		GG	46 (22.5)	1.00 (0.77–1.71) *	3.55 (2.72–4.18)	1.91 (1.36–2.43)	0.94 (0.88–1.00)

Note: * *p* < 0.05, TG, triglyceride; TC, total cholesterol; LDL-C, low-density lipoprotein-cholesterol; HDL-C, high-density lipoprotein-cholesterol.

**Table 5:** Interactions between the ABCA1 genotypes and obesity in patients with low HDL-C.

Variable	*OR*	*95% CI*	*P*	*Adjust OR*	*Adjust 95% CI*		*P*
rs3890182 * obesity	1.409	1.014–1.959	0.041	1.640	1.108	2.427	0.013
rs2275542 * obesity	1.332	1.083–1.639	0.007	1.458	1.141	1.863	0.003
rs1800976 * obesity	1.267	1.072–1.498	0.006	1.356	1.115	1.648	0.002
rs4149313 * obesity	1.373	1.128–1.673	0.002	1.483	1.177	1.869	0.001

Notes: * The interaction of the single nucleotide polymorphism loci and obesity.

### 3.6. Haplotype Analysis

The haplotype analysis results of the six SNPs in ABCA1 gene are shown in Table 6. The global haplotype frequencies were significantly different between the control group and the case group (*p* < 0.001). C-C-C-A-A-G, T-C-C-A-A-A, and T-C-C-A-A-G haplotypes were significantly more frequent in the case group than in the control group, whereas the C-C-C-A-A-A, C-T-G-G-A-A, and T-T-C-G-A-A haplotypes were less frequent in the case group than in the control group (*p* < 0.05).

**Table 6:** Estimated haplotype frequencies of six SNPs in ABCA1 in individuals of the Kazakh nationality with low and normal HDL-C levels.

Haplotype	Case, *n* (%)	Control, *n* (%)	χ^2^	Fisher’s *p*	Pearson’s *p*	OR (95% CI)
C-C-C-A-A-A	5.25 (1.3)	15.20 (03.7)	5.062	0.024	0.024	0.333 (0.122–0.908)
C-C-C-A-A-G	38.73 (9.5)	17.65 (4.3)	8.391	0.004	0.004	2.321 (1.295–4.159)
C-C-C-G-A-G	14.89 (3.6)	21.06 (5.1)	1.163	0.281	0.281	0.689 (0.349–1.361)
C-C-G-A-A-A	16.81 (4.1)	7.75 (1.9)	3.388	0.066	0.066	2.208 (0.931–5.239)
C-C-G-A-A-G	41.88 (10.3)	34.66 (8.4)	0.704	0.401	0.401	1.226 (0.762–1.973)
C-C-G-G-A-G	19.35 (4.7)	11.85 (2.9)	1.831	0.176	0.176	1.657 (0.792–3.468)
C-T-C-G-A-A	59.92 (14.7)	78.83 (19.0)	3.374	0.066	0.066	0.705 (0.485–1.025)
C-T-G-G-A-A	57.08 (14.0)	85.31 (20.6)	7.254	0.007	0.007	0.601 (0.413–0.872)
T-C-C-A-A-A	20.29 (5.0)	2.73 (0.7)	13.708	*p* < 0.001	*p* < 0.001	7.763 (2.174–27.714)
T-C-C-A-A-G	15.50 (3.8)	3.69 (0.9)	7.387	0.007	0.007	4.317 (1.373–13.573)
T-C-C-G-A-G	15.93 (3.9)	8.48 (2.0)	2.296	0.130	0.130	1.905 (0.817–4.441)
T-C-G-A-A-G	19.14 (4.7)	20.30 (4.9)	0.046	0.831	0.831	0.932 (0.490–1.773)
T-T-C-G-A-A	1.89 (0.5)	28.53 (6.9)	24.581	*p* < 0.001	*p* < 0.001	0.061 (0.014–0.267)
T-T-G-G-A-A	31.83 (7.8)	19.82 (4.8)	2.918	0.088	0.088	1.652 (0.924–2.955)

Notes: The haplotype frequencies below 0.03 are not included in the table, and the risk assessment was not performed; global *p* < 0.001; haplotypes of six SNPs in the following order (left to right): rs2275542 (C > T), rs2515602 (T > C), rs1800976 (G > C), rs2230806 (G > A), rs3890182 (G > A), rs41493133 (A > G).

## 4. Discussion

The results of the present study show that the ratio of obese subjects and the levels of TG were higher in cases than in control group, whereas HDL-C was lower. This is probably because the proportion of obesity was higher in cases than in the control group, and elevated TG is one of the major characteristics of dyslipidemia in obesity. Obesity may increase the level of TG in serum by causing insulin resistance and inflammatory reactions [36,37]. In addition, obesity can cause low HDL-C concentrations in serum, another important reason is that obesity-induced hypertriglyceridemia enhances the CETP-mediated interchange of TG from TG-rich lipoproteins to HDL particles and the subsequent TG-enrichment of HDL. Hepatic lipase has greater activity against TG and will thus convert large HDL particles into small HDL particles, which are also cleared more rapidly from the circulation by the kidney, thus reducing the concentration of HDL particles and the levels of HDL-C [38]. Low HDL-C is usually associated with high levels of TC and LDL-C. However, our results show that the levels of TC and LDL-C were higher in controls than in cases, probably because the primary foods in subjects with low HDL-C levels contain high fat products such as wheat, beef, mutton, and dairy product consumption was higher than in individuals with normal HDL-C levels.

ABCA1 gene encodes the protein that regulates the conversion of free cholesterol and lecithin into lipid-poor ApoA1 particles and facilitates the formation of HDL-C and to maintain the stability of the serum of HDL-C level [39,40]. ABCA1 gene mutations may affect its protein transcription and translation, leading to abnormal HDL-C metabolism [15]. Our study showed there was no significant difference between case group and the control group in age or gender, the genotype frequency is consistent with Hardy-Weinberg Equilibrium, with group representative. Frequency distribution of the six SNPs also varied in other different races in the NCBI database (URL:http://www.ncbi.nlm.nih.gov/SNP/snp_ref.cgi?). These results show that there exists significant racial/ethnic variation of allelic frequencies in the ABCA1 gene.

This study found that polymorphism distributions of rs2515602, rs2275542, rs2230806 and rs4149313 were significantly different between the control group and case group. For rs2515602 polymorphism, we observed that compared with T allele, carriers with C allele were more likely to get low HDL-C disease. Carriers with rs2275542 CC genotype were 0.866 times more probable than those with CT genotype to get low HDL-C disease. We also found that compared with rs2230806 G allele, carriers with A allele were more likely to get low HDL-C disease. Moreover, subjects who were carriers with rs4149313 G allele were significantly more likely to get low HDL-C disease. These results suggest that there is association between ABCA1 gene polymorphism and low HDL-C disease.

The relationship of ABCA1 gene polymorphisms and serum lipid levels in humans has been shown in numerous studies, but the data still remain inconsistent. The ABCA1 rs2515602 polymorphism has been found to correlate strongly with HDL-C levels in coronary artery risk development in young adults and TG levels in African Americans [22,41]. The ABCA1 rs2275542 polymorphism was significantly associated with the HDL-C level in the Suita population [23]. ABCA1 rs2230806 polymorphism was significantly associated with the HDL-C level in Egyptians and Asians, G allele was associated with a decrease in HDL-C levels in CAD patients [24,42,43], However, another study shows that G allele was associated with increased HDL-C levels in obese people [29]. On the contrary, an association between ABCA1 rs2230806 polymorphism and HDL-C levels was not observed in young Greek nurses and coronary heart disease patients [44,45]. The ABCA1 rs1800976 polymorphism was associated with the HDL-C level in the Suita population, but no association between ABCA1 rs1800976 genotype and lipid levels was found in another study [23,46]. The ABCA1 rs4149313 and rs3890182 polymorphism were also not associated with the HDL-C level [11,22,26]. In our present study, we showed an association between ABCA1 gene polymorphisms and some plasma lipid levels. The results are in line with some previous studies which supports the association between ABCA1 gene polymorphism and serum lipid levels.

The interactions of ABCA1 gene polymorphisms and obesity on serum HDL-C levels are limited. A study shows that the ABCA1 R230C variant was associated with obesity and low HDL-C levels [47]. A significant negative correlation was observed between the expression of ABCA1 and LDL-C levels in Chinese overweight/obese subjects [48]. Another study showed that carrying the mutant allele of ABCA1 rs2230806 corresponds to a lower level of HDL-C, but HDL-C levels among genotypes in ABCA1 rs4149313 polymorphism were not observed in overweight/obese men [28]. In our present study, we detected interactions between six SNPs of the ABCA1 gene and obesity on low HDL-C disease. These results show that the interactions between rs3890182, rs2275542, rs1800976, and rs4149313 in ABCA1 gene and obesity might increase the risk of low HDL-C disease.

Information about the association of ABCA1 gene haplotype and low HDL-C disease is limited. In this study, we observed that the C-C-C-A-A-G, T-C-C-A-A-A, and T-C-C-A-A-G were significantly more frequent in the case group than in the control group, whereas the C-C-C-A-A-A, C-T-G-G-A-A, and T-T-C-G-A-A haplotypes was less frequent in the subjects with low HDL-C than in the subjects with normal HDL-C level. These findings suggested that the C-C-C-A-A-G, T-C-C-A-A-A, and T-C-C-A-A-G haplotypes may serve as risk factors of low HDL-C disease among Kazakhs, whereas the C-C-C-A-A-A, C-T-G-G-A-A, and T-T-C-G-A-A haplotypes may serve as protective factors of low HDL-C disease among Uyghurs. 

### Study Limitations

There are several potential limitations in our present study. First, the sample size in our study is a bit small. Individuals with rs3890182 AA genotype were not detected in our case group, and the number of subjects with rs3890182 AA genotype in control group was also small. Second, the systolic blood pressure and diastolic blood pressure were high in both groups, and some studies have shown a relationship between hypertension and dyslipidemia [49,50], so while there were no significant differences the case group and the control group, we cannot completely exclude the influence of hypertension on serum lipid levels among genotypes. Third, the interactions of ABCA1 gene polymorphisms and smoking or alcohol drinking on low HDL-C disease were not investigated in our study. Finally, we did not analyzed HDL functionality, however, HDL functionality has been demonstrated to be the cause of the beneficial effects of HDL particles [51]. In summary, it is well known that the HDL-C levels are affected by multiple environmental and genetic factors [52], and their interactions. Although we have discussed the interactions of six ABCA1 SNPs and obesity on low HDL-C disease, there are still many unclear environmental and genetic factors and their interactions that remain to be detected. 

## 5. Conclusions

The present study shows that there was no significant difference in the genotype and allelic frequencies of ABCA1 rs1800976 and rs3890182 polymorphisms between the normal HDL-C group and low HDL-C group. We also found that ABCA1 rs2515602 C allele and rs2230806 A allele carriers were more likely to get low HDL-C disease, carriers with rs2275542 CT genotype were more probable than those with CC genotype to get low HDL-C disease, rs4149313 A allele was less likely to get low HDL-C disease. Besides, we found an association between ABCA1 gene polymorphisms and plasma lipid levels. The C-C-C-A-A-G, T-C-C-A-A-A, and T-C-C-A-A-G haplotypes may serve as risk factors of low HDL-C disease among Kazakhs, the C-C-C-A-A-A, C-T-G-G-A-A, and T-T-C-G-A-A haplotypes may serve as protective factor of low HDL-C disease among Kazakhs. Finally, our results show that the low HDL-C disease was partly influenced by the interactions of ABCA1 gene polymorphisms and obesity.
